# Pure tone discrimination with cochlear implants and filter-band spread

**DOI:** 10.1038/s41598-021-99799-4

**Published:** 2021-10-12

**Authors:** Luise Wagner, Reyhan Altindal, Stefan K. Plontke, Torsten Rahne

**Affiliations:** 1 Department of Otorhinolaryngology, Head and Neck Surgery, University Hospital Halle (Saale), Martin-Luther-University Halle-Wittenberg, Halle (Saale), Germany; 2 Universitätsklinikum Halle (Saale), HNO-Klinik, Ernst-Grube-Str. 40, 06120 Halle (Saale), Germany

**Keywords:** Auditory system, Sensory processing

## Abstract

For many cochlear implant (CI) users, frequency discrimination is still challenging. We studied the effect of frequency differences relative to the electrode frequency bands on pure tone discrimination. A single-center, prospective, controlled, psychoacoustic exploratory study was conducted in a tertiary university referral center. Thirty-four patients with Cochlear Ltd. and MED-EL CIs and 19 age-matched normal-hearing control subjects were included. Two sinusoidal tones were presented with varying frequency differences. The reference tone frequency was chosen according to the center frequency of basal or apical electrodes. Discrimination abilities were psychophysically measured in a three-interval, two-alternative, forced-choice procedure (3I-2AFC) for various CI electrodes. Hit rates were measured, particularly with respect to discrimination abilities at the corner frequency of the electrode frequency-bands. The mean rate of correct decision concerning pitch difference was about 60% for CI users and about 90% for the normal-hearing control group. In CI users, the difference limen was two semitones, while normal-hearing participants detected the difference of one semitone. No influence of the corner frequency of the CI electrodes was found. In CI users, pure tone discrimination seems to be independent of tone positions relative to the corner frequency of the electrode frequency-band. Differences of 2 semitones can be distinguished within one electrode.

## Introduction

In daily life, the ability to discriminate pitch is important for music perception and verbal communication. Different pitches form melodies, and prosodic information in spoken languages carry important information, especially in tonal languages. Pitch is also one of the main perceptual cues for segregating the sources of different concurrent sounds^1^.

Although many cochlear implant (CI) users have good word-recognition capabilities in quiet conditions, detecting differences in pitch and timbre remains difficult^2,3^. CI sound-coding algorithms analyze the envelope of incoming sound and allocate spectral energy to electrode contacts corresponding to the location along the basilar membrane in the cochlea that corresponds to the respective frequency^4–6^. Other algorithms additionally transmit the low-frequency fine structure of the incoming signal to the apical CI electrode contacts^7,8^. Pitch as a perceptual category of “tone height” can be encoded with different cues, such as place pitch (cochlear place of excitation) and temporal pitch (periodicity based). The latter can be divided into rate and modulation pitch for CIs^9–11^.

In most studies investigating music perception in cochlear implant users, complex instrumental tones containing harmonics have been used to investigate pitch perception^13–15^. In discrimination experiments with complex tones, the results range from just-noticeable differences smaller than one semitone in 6 CI users with Cochlear and Advanced Bionics (Valencia, United States) devices stimulated with harmonic complex tones with 15 harmonics^16^, up to 5.7 semitones^1^. Kang et al.^17^ reported large individual variability in pitch-ranking thresholds, ranging from 1 to 8 semitones. Complex tones might stimulate several electrodes simultaneously. To minimize this, sinusoidal tones have been used in some studies. With sinusoidal tones, the stimulation is mainly focused on single electrodes. Depending on the filter settings, stimulation of the adjacent electrodes will also occur. However, the complexity of stimulation patterns remains manageable.

Pogotzelski^18^, investigating 27 Nucleus CI users for frequencies from 494 to 762 Hz, has shown that CI users can discriminate sinusoidal tones only if the frequencies differ by more than 1.3 ± 0.55 semitones. However, the bandwidth of CI electrode contacts is much larger than this reported difference limen. Little is known about whether pitch differences could still be perceived if both tones were allocated to the same frequency band of a single electrode contact or whether pitch discrimination would improve if the tones were allocated to different frequency bands. Pretorius and Hanekom^19^ measured frequency discrimination by presenting sinusoidal tones to 5 Nucleus CI users in an open sound field and claimed that differentiation of tones within one frequency band is possible.

Pure tone discrimination is the simplest psychoacoustic task relating to pitch, so the pure tone discrimination in CIs was extensively tested and analyzed in this study. The electrode arrays of the Nucleus CIs (Cochlear Ltd., Sydney, Australia) are between 14 and 20 mm long and consist of 22 electrode contacts. Electrode arrays of MED-EL CIs (Innsbruck, Austria) are between 15.4 and 26.4 mm in length and consist of 12 electrode contacts. Since the two types of implants transmit signals in a similar frequency range (default settings in the programming software: Cochlear, 188 Hz to 7938 Hz; MED-EL, 70 Hz to 8500 Hz), the resulting frequency bandwidths of the respective electrode contacts are larger in the MED-EL devices. The limited number of available physical frequency channels, i.e., electrode contacts, within the cochleas of CI users explains the reduced spectral resolution compared with normal-hearing people who have thousands of hair cells in each healthy cochlea^12^.

We conducted a prospective psychoacoustic study with Nucleus and MED-EL CI users to compare pure tone discrimination abilities for sinusoidal tones at frequencies within and across electrode contact bandwidths. We hypothesized that discrimination ability improves if the difference between the two tones exceeds the corner frequency of the electrode contact frequency band. Pure tone discrimination was compared between high and low frequencies, that is, between basal and apical CI electrode contacts. Furthermore we hypothesized a difference between manufacturers. MED-EL CI users with larger electrode bandwidths were assumed to have worse semitone discrimination abilities compared to Nucleus CI users.

## Results

All 34 subjects completed the protocol. The mean correctness of answers over all tone pairs, from 0 upto 5 semitones difference, was 58.5% (*SD* 8.4%) for Nucleus CI users and 92.1% (*SD* 12.2%) for the matched NH subjects. In MED-EL CI users, the mean hit rate was 58.5% (*SD* 15.5%), and in the matched NH subjects it was 92.1% (*SD*: 13.6%).

Non-parametric Mann–Whitney *U* tests showed that the hit rate distributions differed between Nucleus CI users and their respective NH control group (*U* = 0, *p* < 0.05, *r* = − 0.82) and between MED-EL CI users and their respective NH control group (*U* = 7.5, *p* < 0.05, *r* = − 0.71). Hit rate distributions between Nucleus CI users and MED-EL CI users (*U* = 137, *ns*, *r* = − 0.02), as well as between the control groups, were not different (*U *= 37, *ns*, *r* = − 0.13).

**Figure 1:**
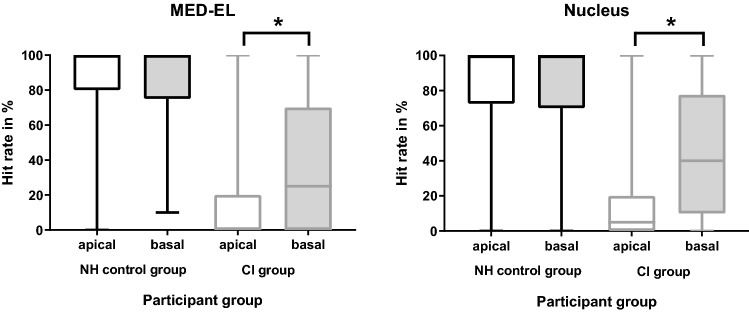
The boxplots show the distribution of the mean hit rates for one semitone distance from the center frequency averaged for lower (apical) and higher (basal) frequencies for MED-EL (left) and Nucleus CIs (right). The median, minimum and maximum are depicted. Asterisks mark statistically significant differences. The difference between apical and basal region for normal-hearing participants was not significant.

Figure 1 shows the hit rates for one-semitone distance when stimulating in the apical or basal cochlear region averaged over all participants of a group. For the control group, no differences were found. The Nucleus control group achieved hit rates of 82.3% (SD 30.3%) for lower tones and 85.2% (SD 28.0%) for higher tones (*p* = 0.234). For the MED-EL control group the hit rates were 78.4% (SD 35.0%) for apical and 84.7% (SD 26.1% for basal stimulation (*p* = 0.089). For the CI users there was a difference between basal and apical stimulation for both electrode types. Nucleus users had hit rates of 17.9% (SD 26.2%) for apical and 37.5% (SD 33.9%) for basal stimulation (*p* < 0.001). MED-EL users also had lower hit rates (15.7%, SD 24.7%) for apical than for basal stimulation (36.1%, SD 35.1%, *p* < 0.001).

**Figure 2:**
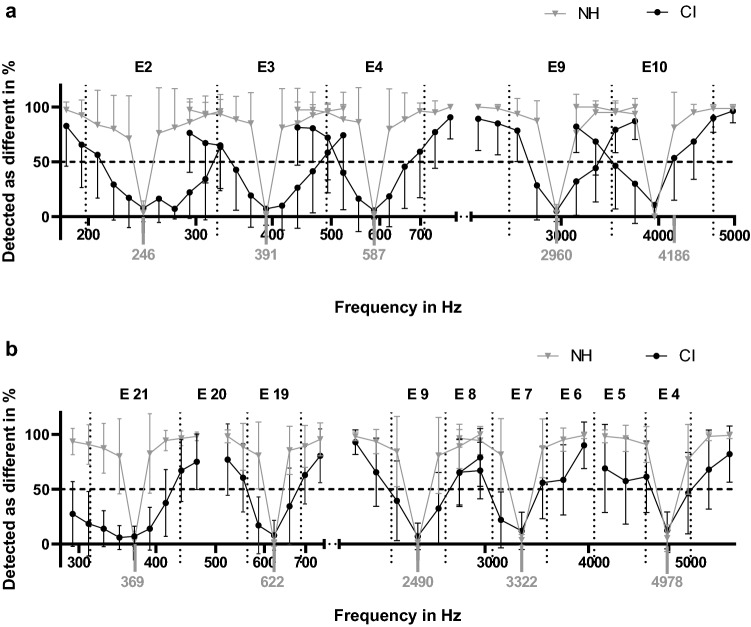
Percentage of tone pairs detected as different as a function of pitch difference. Values are depicted for all used center frequencies (gray values) for MED-EL devices (**a**) and Nucleus devices (**b**). Dotted vertical lines show the corner frequencies of the electrodes (E). Error bars show standard deviations.

Figure 2 shows the hit rates for all CI users and normal-hearing controls as a function of frequency. Normal-hearing subjects were able to discriminate differences of one semitone across all frequencies with hit rates > 71%. The hit rates of CI users were lower than those of NH subjects and increased with frequency difference. Stimuli of equal frequency were rated as identical in all subject groups. Hit rates were higher when center frequencies were allocated to basal electrode bands (mean hit rate of the most basal band of both devices: 33.1%) compared to apical electrode bands (mean hit rate of most apical band: 58.7%). The discrimination functions improved monotonically with pitch difference and did not change abruptly when exceeding the corner frequenciesof the respective electrode contact frequency bands.

For Nucleus CI users, the mean hit rates were compared using a three-way ANOVA for repeated measures using the factors direction of presentation (two levels: up and down), pitch distance (two levels: one semitone or neighbored electrode), and electrode contact (five levels: 21, 19, 9, 7, and 4). The results show that the mean hit rates were significantly affected by direction of presentation (*F*[1,19] = 14.25, *p* < 0.05), pitch distance (*F*[1,19] = 140.39, *p* < 0.001), and electrode contact (*F*[2.51,47.83] = 6.00, *p* < 0.05), as well as by interactions of pitch distance and electrode contact (*F*[4,76] = 5.49, *p* < 0.05), direction of presentation and electrode contact (*F*[4,76] = 4.98), *p* < 0.05), and direction of presentation and pitch distance and electrode contact (*F*[4,76] = 10.72, *p* < 0.001). No effect of the interaction direction of presentation and pitch distance was found (*F*[1,19] = 0.63, *p* = 0.44). Post hoc tests revealed higher mean hit rates for presentation in the upward direction (50.5%, *SD* 3.0%) compared with the presentation from higher to lower frequency (41.5%, *SD* 3.1%, *p* = 0.001). The hit rate for pitch distances exceeding the corner frequencies was higher (59.1%, *SD* 3.2%) than the hit rate for one semitone distance (33.0%, *SD* 2.8%; *p* <  = 0.001). The mean hit rates for the most apical electrode 21 (26.4%, *SD* 4.0%) were significantly lower than those of all the other electrodes (*p*-values < 0.003).

Another three-way ANOVA for repeated measures was calculated for the CI users with MED-EL devices, using factors such as direction of presentation (two levels: up and down), pitch distance (three levels: one semitone, within the same electrode, or neighboring electrode), and electrode contact (five levels: 2, 3, 4, 9, and 10). The results of Mauchly’s tests indicated that no correction for degrees of freedom was necessary. The mean hit rates were significantly affected by pitch distance (*F*[2,26] = 99.38, *p* < 0.001) and electrode contact (*F*[4,52] = 3.19, *p* < 0.05), as well as interactions of direction of presentation and pitch distance (*F*[2,26] = 8.74, *p* < 0.05). Further interactions between direction of presentation and electrode contact (*F*[4,52] = 7.95, *p* < 0.001) and direction of presentation and pitch distance and electrode contact (*F*[8,104] = 3.42, *p* < 0.05) were found. No effect on the hit rate was found for direction of presentation (*F*[1,13] = 0.42, *p* = 0.5) or the interaction of pitch distance and electrode contact (*F*[8,104] = 1.46, *p* = 0.18). Post hoc tests revealed higher hit rates for increasing pitch distance. For a one semitone distance, the mean hit rates were lower (24.2%, *SD* 5.2%) than for the last frequency in the same band (58.8%, *SD* 5.8%; *p* < 0.001), as well as for the first tone in the neighboring band (74.2%, *SD* 4.8%; *p* < 0.001). The comparison of electrode contacts revealed a difference in mean hit rates between the most basal electrode 10 (64.2%, *SD* 6.0%) compared with the two apical electrodes, number 2 (42.3%, *SD* 7.4%; *p* = 0.04) and number 3 (43.6%, *SD* 7.4%; *p* = 0.03).

## Discussion

All CI users and control subjects completed the protocol and were able to discriminate the stimuli. The results show a monotonic increase in hit rate when the tone difference was below the half-frequency bandwidth. It is known from former studies that even when the frequency of the sinusoidal is equal to the center frequency of an electrode contact frequency band, adjacent electrodes are stimulated as well^14^. This co-stimulation of adjacent electrodes increases for tones closer to the corner frequencies of the electrode contact and might therefore be the reason for increasing hit rates with increasing tone difference within the frequency bands. When the tone difference exceeded the corner frequencies (dotted vertical lines in Fig. 2), the continous increase in hit rates persisted. We therefore hypothesize that testing tone discrimination relative to reference tone frequencies at the corner frequency of electrode contacts would result in the same discrimination curves as seen in Fig. 2.

The co-stimulation of adjacent electrodes might be perceived as a discriminatory cue. Nevertheless, this experiment is not just a psychoacoustic measure of the technical parameters, i.e. the band width of the electrode contact.-. This co-stimulation plays a role in discrimination and if it could be reduced by changing coding algorithms, better discrimination could be achieved.

The results show better discrimination of semitones in normal-hearing control subjects as compared with CI users. For a one-semitone pitch difference, the hit rate already exceeded 70% in NH subjects. For larger pitch differences, a ceiling effect was observed. Since this study included NH subjects only as control groups for CI users, we did not measure NH pitch discrimination thresholds, which would probably be below one semitone.

Overall, pure tone discrimination by CI users did not differ between the two devices included in this study. The two CI types differed mainly in their electrode array lengths, the number of electrode contacts within the array, the position within the cochlea relatively to the modiolus or lateral wall, and their frequency bandwidth per electrode contact. The results did not show a general superiority of one device with respect to pitch discrimination, although they stimulate different sites and differ in their bandwidth.

The results show better pitch discrimination at higher frequencies. Stimulation of basal electrodes resulted in steeper discrimination curves, that is, higher hit rates for a one-semitone distance. This might be explained by the stimulation of areas in the cochlea closer to physiological frequency-coding sites^20^. A higher density of spiral ganglion neurons in basal regions might also have contributed to this finding.

The tone discrimination for lower frequencies was worse in Nucleus users compared to MED-EL users. Especially in electrode 21, one tested tail stayed below the chance level. Again, the stimulation of a different site of the cochlea compared with physiological tonotopy might be the reason. The better results of the MED-EL users at lower frequencies can be explained by the additional phase information carried by the fine-structure coding strategy. Another factor is the spread of the electric field which is different forlateral wall compared to precurved electrodes. Especially in the apical region this leads to different patterns. This should be addressed in further studies.

In this study, pure tone differences were analyzed in a same-different psychoacoustic paradigm. Therefore, the results cannot be directly referred to as pitch perception. A level roving was used to avoid focusing on level differences instead of pitch differences. It cannot be ruled out, however, that the CI users additionally rated pitch differences based on other difference dimensions like timbre. Furthermore, it would be incorrect to treatoverall pitch. The ability we investigated was pure tone discrimination. Pitch cues in this study are, on the one hand, the place pitch depending on the stimulated site of the cochlea, and on the other hand, for MED-EL, some phase information in the apical channels.

As pure tones cannot be considered as natural stimuli occuring often in daily life, no general conclusions about hearing with CI in daily life are possible with this experimental setup. However, the results give insights into tone height discrimination abilities in CI users. We showed that pitch discrimination is independent of the actual frequency bandwidths of the electrode contact used for stimulation and therefore independent of the implant model. The effects on comprehensive music perception tasks could be analyzed in further studies. One hypothesis might be that the perception of music pieces is better for higher-pitched tunes since pitch discrimination is easier at higher than at lower frequencies. Hypotheses about the superiority of special keys would depend on the frequency mapping and could also be investigated in further studies.

Many patients still report that, pitch discrimination in CI users is still challenging and we can see that it is still far below the performance of normal hearing subjects. Further improvements are needed to close this gap. Improved CI coding strategies or the use of virtual channels (current-steering) could be developed to improve pitch perception^21,22^.

We conclude that corner frequencies of the electrode contacts and the width of the frequency-band of the respective electrode seem to have no relevant influence on pure tone discrimination in CI users, even in devices that do not use current steering. For the devices used in this study, the number of electrode contacts within the electrode array is not the lone limiting factor for pitch and thus music perception.

## Materials and methods

### Subjects

Thirty-four CI users aged 19–79 years (median 62 years; 17 men, 17 women) and 19 normal-hearing participants aged 18–80 years (median 56 years; 8 men, 11 women) participated in the study and were divided into two groups: one consisted of 20 subjects (age: 55 ± 16 years) provided with Nucleus devices and 11 age-matched normal-hearing controls. The other consisted of 14 subjects (age 65 ± 12 years) provided with MED-EL devices and 8 age-matched normal-hearing controls.

All CI users received their implants at the Halle Hearing and Implant Center of the University Hospital Halle (a tertiary university referral center) and had used their implants for at least 6 months prior to study participation. CI users were included when the word recognition score (WRS) was at least 45% for Freiburg monosyllables at 65 dB sound pressure level (SPL) in quiet. The audio processor settings were checked prior to study participation. All electrodes had to be active and frequency mapping had to be in default setting. In subjects with bilateral implants, all tests were conducted with the CI where the patients eached higher WRS. The stimuli presentation for the control subjects was on the same side as in their respective age-matched CI user. Table 1 provides detailed information about CI subject demographics.

**Table 1:** Demographic and CI-related characteristics of subjects with CI.

Subject no	Sex	Tested CI side	Age at implantation in years	Etiology of hearing loss	Word recognition score*	Duration of CI Use in years	Implant type	Sound processor type	Coding Strategy
113	M	L	52	Sudden hearing loss	90	2	CI512	CP910	ACE
102	M	L	50	Cochlear Schwannoma, Sudden Hearing loss	90	5	CI24RE (CA)	CP810	ACE
116	M	L	67	Chronic Otitis Media	80	5	Mi1000 Flex 28	Opus2	FS4
104	M	R	61	Unknown	80	7	CI24RE (CA)	CP910	ACE
121	F	R	26	Meningitis	60	5	Mi1000 Standard	Opus2	FS4
107	F	L	25	Sudden Hearing Loss	75	2	CI24RE (CA)	CP910	ACE
103	F	R	63	Unknown	70	1	Mi1200 Flex28	Sonnet	FS4
106	F	R	12	Unknown	90	6	SONATAti100 Standard	Sonnet	FS4
117	M	R	72	Unknown	60	1	Mi1200 Flex28	Sonnet	FS4
131	F	L	66	Measles	60	3	Mi1000 Flex28	Opus2	FS4
120	M	L	52	Meningitis	70	4	CI24RE (CA)	CP810	ACE
118	M	R	66	Unknown	65	8	CI24RE (CA)	CP910	ACE
125	F	R	58	Progressive Hearing Loss	95	4	CI24RE (CA)	CP810	ACE
123	F	L	67	Otosclerosis	45	3	Mi1000 Flex Soft	Opus2	FS4
130	M	L	61	Chronic Otitis Media	55	4	Mi1000 Flex28	Opus2	FS4
122	M	R	66	Chronic Otitis Media	95	5	CI24RE (CA)	CP810	ACE
124	M	L	61	Progressive hearing loss	85	18	CI24M	CP910	ACE
127	M	L	71	Otitis Media	60	5	Mi1000 Standard	Sonnet	FSP
128	M	L	45	Cholesteatoma	90	4	Mi1000 Flex28	Rondo	FS4
134	M	R	43	Progressive Hearing Loss	75	8	CI24RE (CA)	CP910	ACE
135	F	R	39	Unknown	70	12	CI512	CP810	ACE
137	F	R	49	Progressive hearing loss	65	9	CI24RE (CA)	CP910	ACE
139	F	R	13	Unknown	70	7	CI512	CP910	ACE
140	F	L	65	Unknown	50	1	Mi1200 Flex28	Sonnet	FS4
142	F	L	59	Progressive Hearing Loss	70	5	Mi1000 Flex28	Opus2	FS4
143	F	L	56	Hearing Loss since childhood	60	5	CI24RE (CA)	CP810	ACE
144	M	L	68	Post-traumatic Deafness	75	10	CI24RE (CA)	CP810	ACE
146	F	R	55	Progressive Hearing Loss	60	3	Mi1000 Flex28	Opus2	FS4
147	M	R	67	Unknown	75	0.5	CI512	CP910	ACE
148	M	R	49	Unknown	75	0.6	CI512	CP910	ACE
149	F	R	62	Unknown	60	1	CI522	CP910	ACE
151	F	R	57	Unknown	55	0.8	CI512	CP910	ACE
152	F	R	61	Ménière's disease	45	6	SONATAti100 Standard	Sonnet	FSP
155	M	L	51	Unknown	80	0.8	CI512	CP910	ACE

*ACE* advanced combination encoder, *CA* contour advance, *F* female, *FSP* fine structure processing, *FS4* fine-structure processing on 4 apical CI electrodes, *L* left, *M* male, *R* right.

*Word Recognition Score: % correct at 65 dB SPL (German Freiburger monosyllable tests).

For control subjects, normal hearing was defined if pure tone hearing thresholds were better than the median hearing threshold according to standard ISO 7029^23^ between frequencies of 0.125 and 8 kHz.

The protocol used in this study was in accordance with the Declaration of Helsinki and was approved by the Ethics Committee of the Martin-Luther-University Halle-Wittenberg (approval number: 2016-151). All subjects provided written informed consent after the procedure was explained to them*.*

### Stimuli and psychoacoustic measurement

Stimuli were generated with MATLAB R2015a software (MathWorks Inc., Natick, Massachusetts, USA) with a sampling frequency of 44.1 kHz. A programmable 2-channel audio attenuator with a resolution of 1 dB (g.PAH, g.tec, Schiedlberg, Austria) was connected to a computer. For CI users, stimuli were presented monaurallydirectly to the audio processor via an audio cable. Prior to the experimental procedure*,* the stimulus intensity was set to the individual’s comfort level based on clinical loudness scaling^24^.

For control subjects, E-A-R tone 3A insert earphones (E-A-R Auditory Systems, Indianapolis, USA) were connected to the attenuator, and stimuli were presented monaurally via the earphones at a sound pressure level of 70 dB.

**Figure 3:**
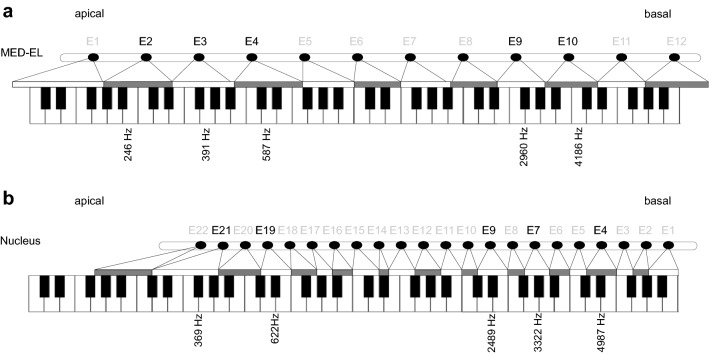
Relationship of electrode contacts (E), frequency bands, and notes (piano keys). (**a**) MED-EL device and (**b**) Nucleus device.

To measure frequency discrimination, some notes of the 12-tone equal temperament system were used for stimulation, matched to the electrode bands of the CI users. The stimuli were pure tones constisting of one sinusoidal. For CI users with Nucleus devices, the basal electrode contacts 4, 7, and 9 and the apical electrode contacts 19 and 21 were used for stimulation. For CI users with MED-EL devices, the basal electrode contacts 9 and 10 and the apical electrode contacts 2, 3, and 4 were used (Fig. 3). The note closest to the respective center frequency was defined as the center note. Tone pairs were generated, including the center tone and a target tone measuring a tone distance of 0 to 5 semitones to the respective center note. The presented tone distances were dependent on the bandwith of the respective electrode. The largest interval included the second semitone of the adjacent electrode contact.

A three-interval two-alternative forced-choice (AFC) same-different procedure was performed in a sound-attenuated chamber.The pattern was always AAB to give the subject the chance to consciously hear the tone necessary for comparison. In every trial, three sinusoidal tone bursts of 500-ms duration separated by a 10-ms interstimulus gap were presented successively. Each burst contained onset and offset amplitude cosine-squared ramps of 30 ms to reduce click sensations. To avoid loudness cues, a level roving with a maximum of 3 dB was applied to the overall bursts. Simultaneously with the presented acoustic signal, numbered buttons were presented on a display to facilitate the task. The corresponding button changed its color from grey to blue as a visual marker. Thus, subjects could visually follow the presentation of the acoustic signals.

After each trial, the subjects’ task was to indicate whether the pitch of the third tone was the same as the pitch of the first and second tone by clicking a button labeled “same” or “different”. The number of correct responses is referred to as the "hit rate". No feedback about the correctness of the decisions was provided. Subjects were not allowed to listen to a trial a second time. However, a subsequent trial was not presented before the subject’s response had been received, allowing the subject enough time to make a decision.

In both groups, each trial (tone pair) was presented 10 times in random order. In total, 370 trials were presented to Nucleus CI users and 510 trials to MED-EL CI users. Breaks were implemented at regular intervals for the participants’ relaxation.

### Data analysis

All statistical analyses were performed using SPSS 25 software (IBM, Ehningen, Germany). The assumption of normality for the hit rates of the CI groups and normal-hearing participants was tested using the Shapiro–Wilk test. The non-normally distributed hit rates for correct pitch discrimination of the CI users and the control groups were compared using Mann–Whitney *U* tests.

Hit rates were compared using an analysis of variance (ANOVA) for repeated measures with the within-subject factors “direction of presentation”, “pitch distance”, and “electrode contact”. Normal distributions of hit rates for each single electrode were assessed using the Shapiro–Wilk tests and visual investigations of Q-Q plots. Alpha was set to 0.05 for all comparisons. For all factors the Mauchly test was calculated. If the test result was significant, the assumption of sphericity was violated and the degrees of freedom were corrected using the Greenhouse–Geisser correction. Post hoc comparisons were done using pairwise comparisons with Bonferroni correction.

Data were plotted with GraphPad Prism 8 (Graphpad Software, Inc., San Diego, USA).

## Abbreviations

AFC: Alternative-forced-choice
CI: Cochlear implant
FAT: Frequency allocation table
NH: Normal hearing
SPL: Sound pressure level
WRS: Word recognition score
